# ESMvis: a tool for visualizing individual Experience Sampling Method (ESM) data

**DOI:** 10.1007/s11136-020-02701-4

**Published:** 2020-11-22

**Authors:** Laura F. Bringmann, Date C. van der Veen, Marieke Wichers, Harriëtte Riese, Gert Stulp

**Affiliations:** 1 Department of Psychometrics and Statistics, University of Groningen, Grote Kruisstraat 2/1, 9712 TS Groningen, The Netherlands; 2 Department of Psychiatry, University Centre Psychiatry, UMCG, Groningen, The Netherlands; 3 Interdisciplinary Center Psychopathology and Emotion Regulation (ICPE), Department of Psychiatry, University Medical Center Groningen (UMCG), University of Groningen, Groningen, The Netherlands; 4 Department of Sociology, University of Groningen/Inter-University Center for Social Science Theory and Methodology (ICS), Groningen, The Netherlands

**Keywords:** Visualization, Experience sampling method, Clinical practice, Intensive longitudinal data, Personalized feedback

## Abstract

**Purpose:**

The experience sampling method (ESM) is used for intensive longitudinal time-series data collection during normal daily life. ESM data give information on momentary affect, activities and (social) context of, for example, patients suffering from mental disorders, and allows for person-specific feedback reports. However, current personalized feedback reports only display a selection of measured variables, and typically involve only summary statistics, thus not reflecting the dynamic fluctuations in affect and its influencing factors. To address this shortcoming, we developed a tool for dynamically visualizing ESM data.

**Methods:**

We introduce a new framework, ESMvis, for giving descriptive feedback, focusing on direct visualization of the dynamic nature of raw data. In this ESM feedback approach, raw ESM data are visualized using R software. We applied ESMvis to data collected for over 52 weeks on a patient diagnosed with an obsessive–compulsive disorder with comorbid depression.

**Results:**

We provided personalized feedback, in which both the overall trajectory and specific time moments were captured in a movie format. Two relapses during the study period could be visually determined, and subsequently confirmed by the therapist. The therapist and patient evaluated ESMvis as an insightful add-on tool to care-as-usual.

**Conclusion:**

ESMvis is a showcase on providing personalized feedback by dynamic visualization of ESM time-series data. Our tool is freely available and adjustable, making it widely applicable. In addition to potential applications in clinical practice, ESMvis can work as an exploratory tool that can lead to new hypotheses and inform more complex statistical techniques.

**Electronic supplementary material:**

The online version of this article (10.1007/s11136-020-02701-4) contains supplementary material, which is available to authorized users.

## Introduction

… sometimes all that is required is a useful visualization …

Spiegelhalter, p. 15 [1]

Increasingly intensive longitudinal data are collected, in which people, such as patients with a mental disorder, are measured daily or multiple times a day for a period of weeks or even months [2–5]. An important motivation for collecting intensive longitudinal data in patients with a mental disorder is to capture psychopathology in its natural daily environment, and by studying it at a micro level, also its dynamics [6]. For example, depressive mood might vary from day to day depending on how much stress is experienced during the days. A further advantage is that when such intensive longitudinal data are used instead of traditional retrospective questionnaires, artefacts such as recall biases can be minimized [3]. Gathering this kind of data are done, for example, using experience sampling methodology (ESM; [7]) or ecological momentary assessment (EMA; [4]). Whereas ESM assesses phenomena such as momentary mood, thoughts, symptoms and context (e.g., social company), EMA is broader and can also include, for instance, physiological assessment and sensor data. ESM and EMA are often used interchangeably; we will use the term ESM hereafter [8].

Importantly, ESM is not just a research tool for studying psychopathology in daily life [9], it is also finding its way to clinical practice. Studying patients intensively over time allows giving context- and individual-specific feedback based on their own ESM data [10]. ESM-based feedback thus allows for detailed idiographic information. This can inform both therapist and patient about moods and thoughts of the patient over the measured period of time, as well as what the patient did, with whom, and where. This unique source of information is currently lacking in standard therapeutic practice, but fits well with the state-of-the-art of case conceptualization [11]. Such ESM-based feedback can furthermore help in tracking down patterns of the individual dynamics of emotions (e.g., how much do emotions fluctuate over time) and the association between those emotions and daily life events [10]. Therefore, ESM-based feedback can help to show possible sources that improve or worsen mood variations over time, and thus not only help to monitor if the patient is doing well or if an intervention is effective, but also give new insights on which behaviours are functional or dysfunctional in daily life. This kind of ESM-based feedback can then guide future therapy interventions. ESM-based feedback is a promising new diagnostic tool empowering both the patient as well as the therapist [12].

The additional benefit of ESM-based feedback was shown in a pioneering randomized control trial of Kramer et al. [13]. In this study, patients with a depressive disorder either just filled-out an ESM questionnaire or also received individual-tailored feedback based on their own ESM data. It appeared that patients with a depressive disorder who received ESM-derived feedback versus depressed patients who did not receive such feedback had a clinically relevant decrease in their depressive symptoms. This suggests that personalized ESM-derived feedback can be beneficial. More generally, it highlights that ESM-derived feedback can be a useful addition to the treatment for patients with a depressive disorder.

ESM-derived feedback comes in many forms. This can vary from real-time advice or interactive feedback [14, 15] to a feedback report containing analyses or summary statistics summarizing a certain period in which ESM data were gathered [10]. Regarding real-time advice, Bauer et al. [14], for example, gave their patients feedback in real-time through person-tailored text messages promoting, for instance, social support. Similarly, Hareva et al. [15] continuously analyzed ESM data of a patient after every four data points to check if his values were below a beforehand set threshold. In case ESM scores were below the threshold the patient would receive real-time advice by an e-mail containing the message: “Let’s take a rest”.

Other studies have given ESM-based feedback through a report. Such a report could be given face-to-face or online [16, 17]. Several studies provided verbal feedback in a face-to-face fashion that was complemented with visual summaries of weekly affect: a pie chart showing, for instance, the amount of time spent on activities (e.g., eating and drinking or household activities) and the amount of positive affect during these different contexts or activities. After several weeks, feedback furthermore included bar graphs indicating changes in weekly affect level and depressive complaints throughout the ESM study [13, 16]. Other popular ways of giving descriptive feedback are means and line graphs of a single or several variables showing, for instance, if there is a trend in the data [18]. Unique is the *N* = 1 study of Groot [19], where visualizations of patterns of emotions over time were combined with information on specific physical activities (e.g., running). Van Roekel et al. [20] gave similar face-to-face feedback as in previous studies, but additionally included specific suggestions on how participants could increase feelings of pleasure in their lives. Furthermore, individual feedback also contained comparison of one’s own scores to a norm group. In a large, still ongoing crowdsourcing study “How Nuts are the Dutch” the ESM-based feedback was similar in style as in the aforementioned studies, but was only given online, and not in face-to-face [17].

Recent studies often go beyond descriptive statistics and try to additionally offer feedback based on inferential statistics [16, 20–23]. Such inferential statistics mostly takes the form of Vector AutoRegressive (VAR) modelling, with which one can detect, for example, whether certain activities at a given time point lead to more or less positive affect at a later time point [21, 24, 25]. Inspired by the network approach, such models are presented and visualized as personalized networks [26, 27]. According to the network approach, causal interactions between variables such as symptoms should be the focus of study in understanding what mental disorders are and how these disorders develop [28–30]. The idea behind providing personalized networks then is that the potentially causal relationships between momentary self-reported affect states, context and behaviours could provide possible intervention targets for therapy [18].

Although increasing the complexity of ESM-based feedback may hold great promise for getting the most out of ESM data, the usefulness of this approach for patients is still unclear. The interpretation of VAR-based network models is highly depended on the exact network model used and different choices regarding a VAR based network model can lead to widely different recommendations for the patient [31, 32]. This not only results in conflicting advice on where and how to perform further interventions, but also in the additional complication that at the moment we do not have well developed tools to evaluate these different outcomes. Furthermore, the complexity of what is exactly modelled makes it more difficult for patients and therapists to fully understand the ESM-derived feedback.

As such complex statistical methods still face many issues that need to be overcome [33], in this paper we turn our attention to further developing descriptive measures for personalized feedback. We introduce a new framework for giving descriptive feedback. Currently used descriptive statistical measures, such as histograms, only present a summary of the data and do not reflect the dynamic fluctuations in affect and factors that influence it. To address this shortcoming, we give back to the patient and therapist the data as it was filled-out by the patient. In this new ESM visualization approach, or ESMvis, the raw ESM data can be visualized in a dynamic way using the freely available software *R* [34]*.* Thus, instead of using summary descriptives such as averages, or only showing selected variables that were measured, this visualization technique presents both the overall trajectory and the specific time moments in a movie format. This includes not only quantitative measures, such as how much a patient scored on a certain variable, but also qualitative information, such as written information about an experienced (un)pleasant event.

In order to showcase this new visualization approach, we illustrate all aspects of the visualization with ESM data of a clinical patient, who monitored herself over 1 year as part of a relapse prevention plan. We show that the method was experienced as insightful both by the therapist and the patient, and can potentially function as an add-on tool to care-as-usual. Both the data and the R-code is freely accessible and links will be provided in the “Methods” section.

## Methods

### Patient and informed consent

The participant is a 31-year-old woman suffering from an obsessive–compulsive disorder (OCD) with comorbid depressive symptoms for twelve years. She has been treated, following the guidelines, with medication, polyclinical and clinical treatment programmes. At the time of this study she was following a cognitive behavioural-based day-clinical programme. Her obsessions consist of intrusions of harming herself and others or losing control over her behaviour and shaming herself. Her compulsions consist of just-right and checking rituals. At a personal level she has a strong belief she is failing when she was not able to cope with her anxiety by herself. She has a bright and curious personality and a healthy social life, a boyfriend and supportive parents. The protocol used was submitted to the ethical review board of the UMCG, who confirmed that formal assessment was not required (METc No. 2015/140). Prior to participation, the patient was fully informed about the study, after which she gave written informed consent. The study was planned to go on for as long as the patient found the ESM data collection to be useful.

### ESM questionnaire and procedure

An individually tailored ESM questionnaire originated from her written relapse-prevention plan containing questions that concerned both momentary assessments as well as retrospective assessments of events that occurred since the last measurement point (see Box 1 for the full ESM questionnaire). Notice that the questionnaire contains both positive (e.g., I feel like doing something fun) and negative variables (e.g., I am afraid to lose control), which can also be seen as resilience-related and OCD-related variables, respectively. The patient was interested in the patterns of how the variables develop over time, for example when transitioning to a relapse. The therapist was also expecting to find patterns that could function as early warnings signals for relapse.

Filling out the questionnaire took circa 1 to 3 min. The questionnaire was offered three times a day at fixed time points. The exact times when the questionnaire was sent out (9:00, 15:00 and 21:00 o’clock) were adjusted to the patient’s daily rhythm, with the last measurement occurring circa 30 min before going to bed. The instruction for the patient was to fill out the questionnaire immediately, or otherwise within 15 min after receiving the notification (beep). After 30 min a reminder was sent, and after 60 min the link was deactivated. Data were gathered with a secured server system (RoQua, [35]). With this system, text messages with links to online questionnaires were sent to the respondent’s smartphone. She filled out the questionnaire over 1 year, starting in March 2017.

**Box 1** The ESM questionnaire that was filled out by the patient on a smartphone. Note that question 2 was presented in the form “Are you sad”, but based on discussions between the therapist and the patient on the exact connotations of the items, it was found that the patient also interprets this item as feeling “meaningless” or “useless”.

**Table:** 

Scoring is done on a scale with a slider from 0 to 100 (unless indicated otherwise)
The first set of questions concerns momentary assessments
1. I am afraid to lose control [Scale = (not at all – very much)]
2. I feel sad / useless / meaningless [Scale = (not at all – very much)]
3. How convincing are the intrusions [Scale = (not at all – very much)]
4. I can encourage myself [Scale = (not at all – very much)]
5. I feel like doing something fun [Scale = (not at all – very much)]
6. I have the feeling that “I can do this” [Scale = (not at all – very much)]
7. Are you in company? [Yes / No]
8. How afraid are you of being alone? [Scale = (not at all – very much)]
The next set of questions concerns occurrences since the last measurement point
9. How often have you been in contact with someone you feel safe with? [Score: 0–1–2–3–4]
10. How often have you thought of contacting someone you feel safe with? [Scale = (not at all – very often)]
11. How strong was your inclination to cancel appointments? [Scale = (not at all – very much)]
12. Have you actually cancelled appointments? [Yes / No]
13. Have you laid on the couch or in bed since the last measurement point? [Scale = (not at all – very often)]
14. Have you slept since the last measurement point? [Yes / No]
15. If yes: How did you sleep? [Scale = (badly – very well)]
16. Did you leave the house? [Scale = (not at all – very often)]
17. How did you eat? [Scale = (not at all – very well)]
18. Have you avoided everyday things? [Scale = (not at all – very much)]
19. Have you done useful (important) things? [Scale = (not at all – very much)]
20. Have you enjoyed your activities? [Scale = (not at all – very much)]
21. Since the last measurement point, have you experienced any (un)pleasant everyday occurrences?
22. Yes, something pleasant: How pleasant was this experience? [Scale = (not at all – very much)]
23. Yes, something unpleasant: How unpleasant was this experience? [Scale = (not at all – very much)]
If you want, you can add comments here:
[space for comments]
Concluding sentence:
‘Thank you very much for filling out the questionnaire, and do not forget to charge the battery of your smartphone.’

### ESMvis

ESMvis is a method for visualizing ESM data of a single individual with the software *R* [34]. It allows visualizing all of the data across time, data from a single measurement, or a combination of the two. This means that insights from overall patterns can be easily verified and cross-checked with data on the measurement level and vice versa. Central to ESMvis is the *circle figure*, in which all data from a single measurement is presented at once. This section is a walkthrough of the package and its different options. For more detailed information on the *R-*code, please see the file *Vignette-QLR.html* in the Supplementary Material, in which the figures produced in this paper are explained in detail and the code to produce the figures is provided. Throughout the text we will refer to the different sections of this html file. All data and code to produce the visualizations, video, and Shiny app can also be found at the following link: https://doi.org/10.34894/RMJHEJ.

#### Quick overview: variation of all variables (boxplots)

**Fig. 1:**
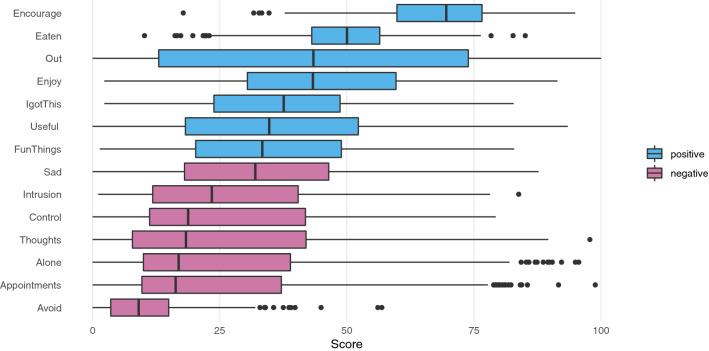
Boxplots for resilience- ("positive") and OCD-related ("negative'") variables, ordered on median

The first part of the ESMvis tool is meant merely for researchers to get a quick overview of the variation that exists in the different variables measured through boxplots (see “Boxplot” section in the html file). These boxplots contain the median (thick line), lines that represent the first and third quartile between which 50% of the data lie (the box), whiskers (the lowest/highest point within 1.5 times the interquartile range from the first/third quartile, respectively), and points/dots to signify outliers. Different colours are used in the plot to signify positive (blue) and negative (pink) variables (i.e., resilience-related and OCD-related variables, respectively). Decisions on the category to which a variable belongs were made together with the therapists who developed the questionnaire. Note that the event variables or variables that were measured on a Likert scale and were not present at every time point (see the next section) are not shown in the boxplots (Fig. 1).

#### Line figure: overall view

**Fig. 2:**
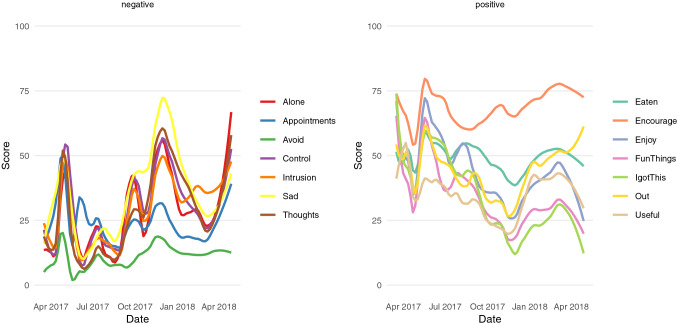
Overview of the change over time for all resilience- and OCD-related variables. Lines are smoothed LOESS-curves with a kernel bandwidth of 0.2

In order to get an overview of the ESM data over time, line figures that represent the resilience-related (positive) and OCD-related (negative) variables over time are made (see “Create time line” section in the html file). In the left panel, fluctuations in negative variables are visualized over the period March 2017 to April 2018, and in the right panel fluctuations of positive variables. These lines do not represent the underlying raw data but instead are smoothed LOESS-curves (Fig. 2). Although the smoothing is set rather low, it is possible that the data are over-smoothed and important features of the data are concealed. This can happen, for instance, when the data have a lot of sudden jumps (see for instance, Wood 2004). Therefore, it is important to vary the smoothing parameter and examine the raw data to better understand the underlying patterns. Showing the raw data over time without smoothing is also possible, but in this case resulted in less intuitive visualizations.

#### Circle figure: all data at once

**Fig. 3:**
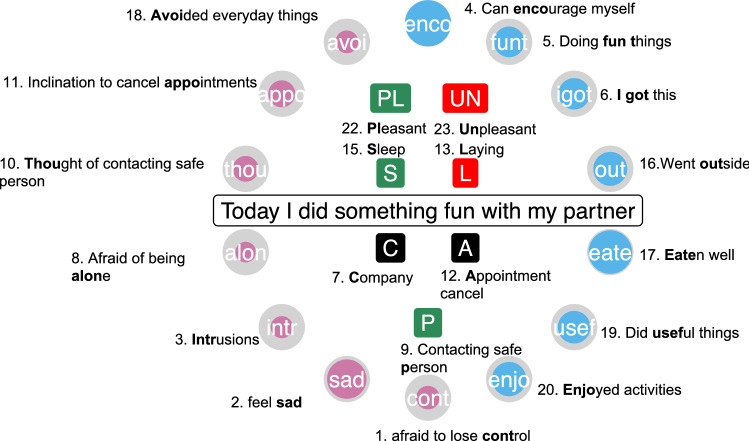
The legend for the circle figure that is always included when the therapist gives the feedback to the patient (If a node is grey or a label in the middle is absent, this can mean either that the patient did not fill it in, or that indeed the variable or event was indicated to be zero or absent, respectively.)

To be able to see all the data collected at a single measurement (including events, remarks, and variables measured on a Likert scale), we developed a circle figure (see “Create circles” section in the html file). In Fig. 3, all variables of the ESM questionnaire that the patient scored are shown. The circles in this figure represent the negative (OCD-related) and positive (resilience-related) variables visualized before in the boxplot and timeline plot, but now for a single time point. A completely grey circle means that the patient scored zero on the specific variable, and if the circle is completely filled with a colour, the patient scored 100 on that specific variable. Colours match those in the boxplot. For example, the patient reported that she could encourage herself very well and her score of 100 is reflected in the blue variable in the top of Fig. 3. The middle part contains all the other variables: if there was an (un)pleasant event and how (un)pleasant it was, whether the patient slept and how the sleep quality was, if and how much the patient has been laying down on a couch or bed, if the patient has had company or not, if she cancelled an appointment, and whether she has contacted a person she feels safe with. For example, in case of Sleep Quality, she first scored if she had slept (if yes, the S symbol will appear), and in a second step she indicated the sleep quality (the darker the green the higher the score).

**Fig. 4:**
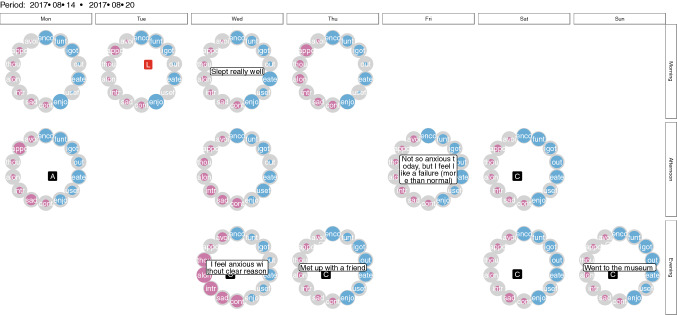
Weekdays (horizontal, on top) and morning, afternoon, and evening (vertical, right side) circle figures of the ESM questionnaire that was sent out three times a day

Instead of just one circle figure or time point, it is also possible to view several measurements at once, for example, over 1 week (see Fig. 4) or longer. This gives an immediate idea of how well the patient felt in that time period. In the Supplementary Material (Poster_all_responses.pdf), we also show a poster of all measurement points including all circle figures over time, which allow extracting better and worse periods. Pink periods indicate more difficult periods than blue periods. As shown in Figs. 3 and 4, qualitative information can also be represented in the circle figure by simply adding a text box with the commentary for that specific time point.

#### ESMvis movie and Shiny app

**Fig. 5:**
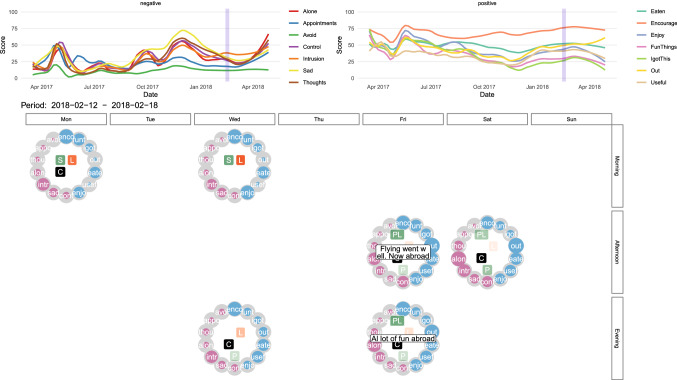
A slice from the ‘movie’ format visualization where the line plots and the circle figures can be viewed at the same time

It can also be insightful to combine a ‘macro’ perspective of the changes over time with a ‘micro’ perspective on the measurements. In order to allow for this, we have included a feature to combine line plots with weekly circle figures. In the line plot, the week that is under scrutiny is indicated with a blue overlay, and in the circle figures, a snapshot of that particular week is shown. Online a ‘movie’ can be found in which each week is visualized for the entire duration of the study (see http://shiny.gmw.rug.nl/QLR/video/). We also developed a *Shiny app* (see http://shiny.gmw.rug.nl/QLR/shiny/) [36], which allows the opportunity to click on or off certain functions of the ESMvis, for example, if one wants to only see the circle figures or leave out the commentaries of the patient (Fig. 5).

## Results

### General description of the variables

Results in Fig. 1 clarify two things: (1) the patient typically scores higher on positive variables than on negative variables and (2) in general she seems to score on all variables rather low with scores often not higher than 80 and frequently below 50. The highest scores are on the variable “I can encourage myself”. The lowest scores are for the variable “avoiding everyday things”. Furthermore, for most variables there is large variation in scores (e.g., “Out” indicating if she left the house) or they have outliers (e.g., “Alone” indicating if she is afraid of being alone). In order to get a better idea of the dynamics, for instance, when and in what kind of contexts scores on the variable “Alone” were high, we will now discuss the results of the ESMvis movie.

### Results of the ESMvis

Based on the ESMvis movie, focusing on the negative variables, it seems there are two setbacks in the year in which the patient measured herself (see http://shiny.gmw.rug.nl/QLR/video/). These are around the end of April 2017 and end of September 2017. This last period intensifies even more into a bigger relapse in December the same year. Importantly, these peaks corresponded with the relapses, reported independently from the ESM data, by the therapist (namely: 20-04-2017 and 25-09-2017). Regarding the first relapse, it is clear that already in March there are time points in which the patient reports negative events (such as the first measurement on Thursday in March) or even whole days (e.g., the last weekend of March). The pink or negative scores become more constant and higher over time until it ebbs away in the mid of May.

A finding that the therapist and patient found interesting is that “sadness” and “cancelling appointments” seem to be increasing in scores the earliest before the first relapse. Before the second relapse, “sadness” is again clearly increasing, but not “cancelling appointments”. This was a new insight for the therapist, who expected “cancelling appointments” or OCD-related symptoms to be the key early warning signals.

Another prominent finding that only becomes visible when the macro- (the line plots) and the micro-perspectives (the circle figures) are combined is that even though the patient often wants to cancel an appointment (as is indicated by the *Appo* node in the circle), she often does not actually cancel the appointment (indicated by the square with a *c* in the middle of the circle; see also Fig. 3). Regarding “afraid of being alone” (i.e., the *Alon* node) we see that extreme outliers (higher scores than on average) especially turn up in periods when she experiences a relapse. It is also an advantage that the ESMvis tool shows clearly when the patient did not fill in the ESM questionnaire, such as the period around mid-May.

Furthermore, around August the patient starts to fill-out qualitative commentaries. For example, mid-September the patient states: “A lot of anxiety, I feel like I am back at square one, I have to travel abroad soon.” This gives immediate insights into what the unpleasant events were that day and thus gives context to why she scored very high on many of the negative variables at that moment. Thus, it not only shows which symptoms a patient has but also the content of these emotions or worries: what the patient is sad or worries about. Furthermore, these qualitative comments show with whom she was at a specific time point revealing, for instance, that her parents are very supportive when she has a setback.

### Feedback moment together with patient and therapist

On the 21st of March 2018 the therapist, patient and one of the ESMvis developers had an informal feedback moment together to discuss the ESMvis results. A poster of all circle figures was printed out (see Poster_all_responses.pdf in the Supplementary Material) and the ESMvis movie was shown. We discussed the patterns that we detected in the ESMvis movie as described above. The patterns and the visualization were experienced as insightful by the patient. The patient and therapist not only recognized patterns that they had expected to find, but also discovered new interesting patterns, such as the clear increase in “sadness” before both relapses.

## Discussion

We have provided a first demonstration of a new tool for the dynamic visualization of raw ESM data. The feedback was experienced as insightful by both the therapist and the patient, indicating patterns that could potentially help in future treatment of the patient. Our tool is freely available, adjustable, and easy to use, making it widely applicable to different kinds of ESM data. In addition to potential applications in clinical practice, gaining insights into the data is also a crucial first step before running more complex analyses. As such, ESMvis can work as an exploratory tool that can lead to new hypotheses, and in the end, inform more complex techniques [37, 38].

One of the main advantages of ESMvis is that, in contrast to most statistical methods such as VAR, there are no restrictions regarding the number of time points that are needed. Any ESM dataset, short or long, can be visualized and reported back to the patient and therapist. Furthermore, not only the raw data but also the missingness is explicitly represented, giving an immediate grasp on how much missingness there is and when. In addition, there is a complete representation of all the data, including the context in which the emotions fluctuate, and the qualitative information and commentaries that the patient fills out. ESMvis is a comprehensive tool for visualization of complex time-series data collected for a long period (e.g., weeks, months) during daily life of an individual patient.

The wealth of information in ESM data, however, can also lead to problems for ESMvis. For example, when many (e.g., 10) variables are represented in the line plots, it will be difficult to discern them from each other. Fortunately, this issue is largely solved using the Shiny app, in which you can click some information on and off (such as variables in the line plots, or the commentaries in the circle figure), or zoom in to a certain time period if wanted. Additionally, adjusting the ESMvis for other studies with different setups might require extensive recoding. First, for new ESM questionnaires, one has to always decide upfront what will be classified as positive, negative and event variables. Second, when the data form is not exactly the same as in the current study (e.g., large number of variables with different scales, multiple events), the current code needs to be adjusted requiring some further programming. Thus, at the moment, ESMvis is not a standalone tool for clinical practice.

Another limitation is that even though ESMvis was experienced as insightful by the therapist and patient, a more systematic way of studying its usefulness is needed to confirm the clinical relevance of the tool. This could take the form of a randomized controlled trial or a qualitative study consisting of structured interviews with therapists using the tool [39]. Relatedly, more research is needed that explicitly focuses on the clinical benefit of ESM feedback for the patient over and above just filling in the ESM questionnaires (along the lines of [13, 16, 40]).

To conclude, we hope to have shown that ESMvis can be used in addition to current personalized feedback methods, and can be informative for researchers, therapists and patients to get a full grasp of the complexity of ESM data and daily life in general.

## Electronic supplementary material

Below is the link to the electronic supplementary material.

(PDF 1260 kb)

(CSV 110 kb)
