# Supplementary figures and images for: ESMvis: a tool for visualizing individual Experience Sampling Method (ESM) data
Source: Qual Life Res. 2020 Nov 22;30(11):3179–88. doi: 10.1007/s11136-020-02701-4 (PMC8528801; doi:10.1007/s11136-020-02701-4)

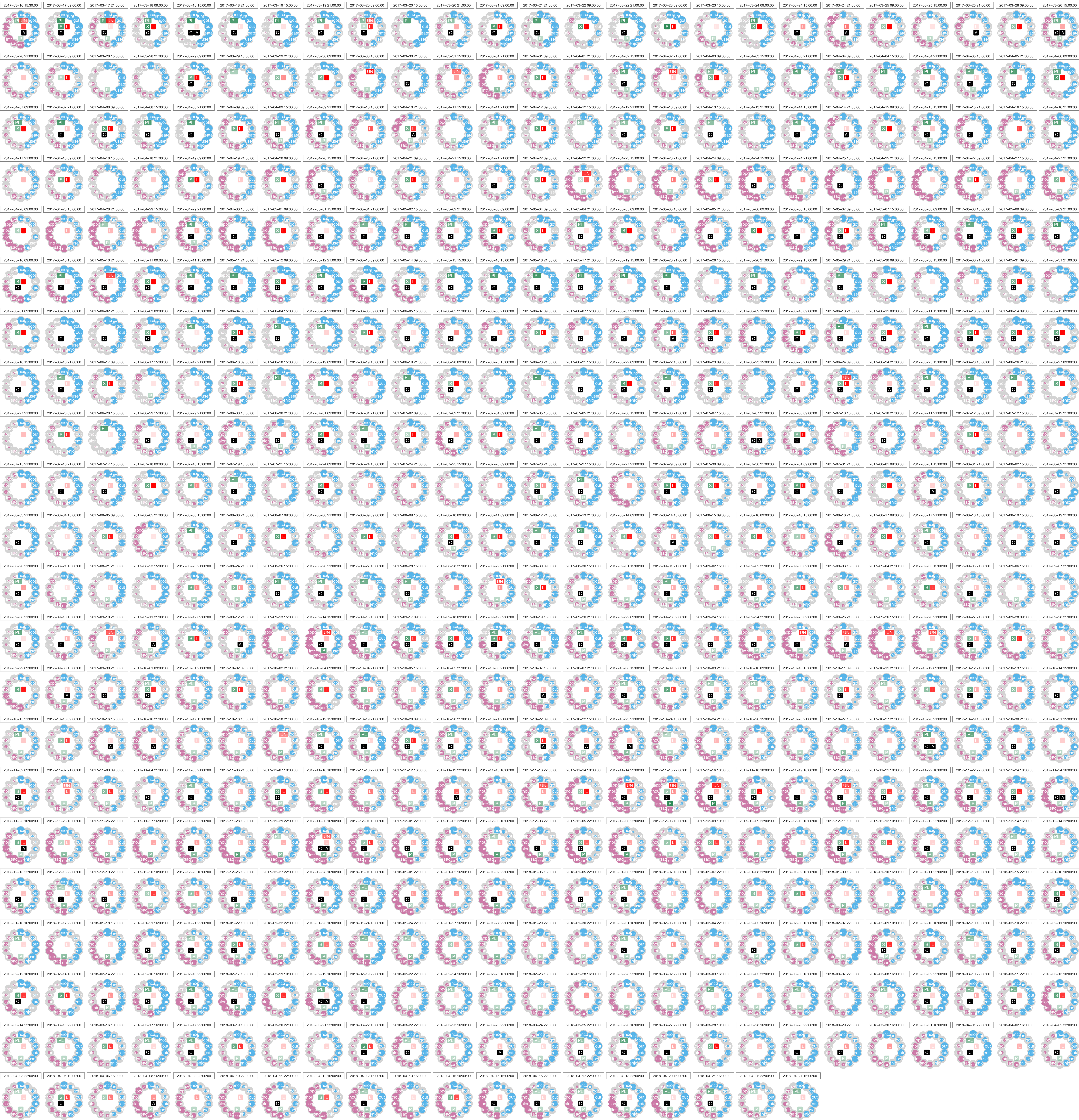

Supplement: Supplementary file 1 — (PDF 1260 kb) [file 11136_2020_2701_MOESM1_ESM.pdf]
